# The Role of Social Cognition Abilities in Parkinson's Disease in the Era of COVID-19 Emergency

**DOI:** 10.3389/fpsyg.2021.571991

**Published:** 2021-03-30

**Authors:** Alessandra Dodich, Costanza Papagno, Luca Turella, Claudia Meli, Francesca Zappini, Pamela Narduzzi, Alessandro Gober, Enrica Pierotti, Marika Falla

**Affiliations:** ^1^Center for Mind/Brain Sciences - CIMeC, University of Trento, Rovereto, Italy; ^2^Psychology Department, Università degli Studi di Milano-Bicocca, Milan, Italy

**Keywords:** social cognition, COVID-19, Parkinson's Disease, caregivers, cognition

## Abstract

**Introduction:** Parkinson's Disease (PD) is characterized by motor and non-motor symptoms, among which deficits in social cognition might affect ~20% of patients. This study aims to evaluate the role of social cognitive abilities in the perceived impact of COVID-19 emergency, and the effects of lockdown measures on patients' social network and caregivers' burden.

**Methods:** Fourteen PD patients performed a neuropsychological battery including sociocognitive tasks before the introduction of COVID-19 restrictive measures (i.e., social distancing and isolation). A structured interview through an online platform was performed in the last 2 weeks of the first lockdown phase to assess patients' health status, perception of COVID-19 emergency, changes in caregivers' burden, and patients' social isolation. Non-parametric analyses were performed to evaluate the association between social skills and patients' COVID-19 perception, as well as the effects of restrictive measures.

**Results:** At baseline evaluation, half of the PD patients showed sociocognitive dysfunctions, mainly on mentalizing abilities. Patients with impaired social cognition skills showed a significantly lower concern on the possible effects of COVID-19 on their health. Caregiver burden and patients' social network remained stable during the lockdown.

**Conclusion:** These preliminary results underline that PD sociocognitive dysfunctions might affect patients' abilities to estimate the effects of COVID-19 infection. However, the lack of a significant increase in caregivers' burden and social isolation suggests, in our sample, a good coping to COVID-19 emergency. Since COVID-19 pandemic can have direct and indirect severe consequences in patients with PD, the development of educational and preventive programs is recommended.

## Introduction

Since the end of 2019, the world has been overwhelmed by the spread of a new coronavirus (SARS-CoV-2), which forced the World Health Organization to declare the state of pandemic in March 2020. This action has been paralleled by the introduction in many countries of restrictive measures, such as social distancing, quarantine, and massive closure of commercial and productive activities, with a substantial effect on people habits and routines. To prevent the spreading of the virus and the high risk of the health-care system overwhelming, the Italian government introduced emergency security measures on March 9, 2020, including closed borders, social distancing, and isolation. With the progressive control of the emergency in terms of reduced rate of positive cases, hospital admissions, and patients requiring intensive care, less stringent measures were introduced. Overall, the Italian lockdown included restrictive measures that lasted from March 9, 2020 to May 3, 2020. However, the long-lasting period of quarantine may have led to important consequences in healthy subjects (Cerami et al., 2020) as well as in patients living with a chronic disease such as Parkinson's Disease (PD).

The restricted access to health care, physical inactivity, and psychosocial effects (Prasad et al., 2020) could potentially worsen motor and non-motor symptoms characterizing the disease and resulting from nigrostriatal dopamine depletion. Among these, cognitive symptoms might include executive, visuospatial, and social cognitive dysfunctions (Robbins and Cools, 2014). Notably, extensive literature on PD reports significant deficits in different facets of the social cognition such as emotion recognition (e.g., Baggio et al., 2012; Mattavelli et al., 2020), theory of mind (e.g., Bodden et al., 2010), and empathy (Martinez et al., 2018). Overall, social skills play a crucial role in detecting and predicting actions, intentions, and emotions on the basis of individual knowledge (Barnes-Holmes et al., 2004), and they have been previously related to the individual engagement in recommended precautions during a health crisis (Puterman et al., 2009). An impairment in these abilities in the context of COVID-19 quarantine may thus lead to severe consequences, such as situational misinterpretations and increased conflicts in social and familial relationships.

Social cognition deficits contribute to characterize PD clinical picture together with other motor and non-motor symptoms. With the progression of the disease, PD patients might tend to move from a public into a private world to mask these symptoms, with a significant and progressive reduction in their social engagement due to stigmatization (Maffoni et al., 2017). Social isolation represents one of the major risk factors for the development of cognitive decline and dementia (Livingston et al., 2017), and specifically in PD social, support has been found as one of the major variables in positively affecting daily living (Ambrosio et al., 2019). In this sense, informal caregivers have a key role in practical and psychological support for PD patients. However, this role exposes them to changes in emotional and physical health, social life, and financial status (Martinez-Martin et al., 2012). Despite an emerging body of literature on PD care during the COVID-19 emergency (Papa et al., 2020; Prasad et al., 2020; Salari et al., 2020; Shalash et al., 2020), very little is known about caregivers' experiences. Quarantine and lockdown measures significantly forced them to deal with changes in their daily routines, including personal, family, and patients' management. While recent evidence showed in PD carers higher anxiety levels related to COVID-19 emergency compared to controls (Salari et al., 2020), it is still unclear whether the restrictive measures affected caregivers' well-being.

The aim of this preliminary study is thus to explore the effects of social cognition deficits in the perception of COVID-19 emergency in PD patients as well as their relationship with caregivers' burden. Besides, we investigated possible changes in patients' perceptions of social support and in caregiver well-being through an online interview performed in the last 2 weeks of the first lockdown phase in Italy. We hypothesized that social cognition deficits in PD might affect patients' perceptions of the COVID-19 emergency and that COVID-19 containment could possibly increase PD patients' perceived social isolation and caregivers' burden.

## Materials and Methods

### Subjects

Fourteen PD patients were included in the context of the longitudinal project “Study of the neural bases underlying the beneficial effects of physical activity in Parkinson's disease.” Patients were recruited from the Centre for Neurocognitive Rehabilitation (CeRiN) in Rovereto, Italy. Inclusion criteria were diagnosis of idiopathic PD based on the criteria of the International Movement Disorder Society (Postuma et al., 2015), Hoehn and Yahr scale ≤3 (Hoehn and Yahr, 1967); absence of dementia or other significant psychiatric or neurological disorders; basic computer knowledge and Internet access. All subjects underwent a baseline clinical evaluation by experienced neurologists and neuropsychologists between January and February 2020, and a follow-up assessment through an online platform between April 20 and May 3, 2020. Patients were assessed during the “ON” state, and data on the motor symptoms through the Unified Parkinson's Disease Rating Scale (MDS-UPDRS) part III (Goetz et al., 2008) were collected. Caregivers were interviewed at both baseline and follow-up. Demographics and clinical data for the 14 PD patients are shown in Table 1. Eleven patients were characterized by a tremor-dominant phenotype and three patients by a postural instability and gait disturbances phenotype. Mean UPDRS-part III score was 15.5 ± 5.8. All patients were treated at the time through antiparkinsonian drugs, that is (levodopa, dopamine-agonists, MAO-, and COMT-inhibitors). Patients' caregivers included first-degree relatives (caregiver-patient relationship: consort *n* = 12, offspring *n* = 2; caregiver civil status: married *n* = 13, single *n* = 1; male/female: 6/8; average age in years = 60.2 ± 13.4, average years of education = 13.5 ± 4.6).

**Table 1:** Demographics and clinical profile of the enrolled sample.

	**Mean ± SD**	**Cut-off score**	**Impaired performance (borderline)**
**DEMOGRAPHICS**			
Sex (number of M/F)	7/7		
Age in years	55.5 ± 8.7	-	-
Years of education	13.4 ± 4.8	-	-
**CLINICAL**			
UPDRS III	15.5 ± 5.8	-	-
Geriatric depression scale	8.1 ± 7.2	>9^*^	5 mild, 1 severe
Disease duration (years)	5.7 ± 4.1	-	-
**NEUROPSYCHOLOGICAL**			
MoCA (0–30)	24.4 ± 2.6	<17.363	0 (1)
Digit forward (0–8)	6.1 ± 0.9	<4.26	0 (0)
Digit backward (0–8)	4.3 ± 0.9	<2.65	0 (1)
Corsi block tapping task (0–9)	5.1 ± 1.1	<3.46	1 (3)
Rey Auditory Verbal Learning immediate recall (0–75)	46.0 ± 9.3	≤28.52	0 (0)
Rey Auditory Verbal Learning delayed recall (0–15)	9.4 ± 2.4	≤4.68	0 (0)
Rey-Osterrieth complex figure recall (0–36)	16.3 ± 6.0	<9.47	0 (2)
Trial making test A	44.4 ± 15.9	≥94	0 (0)
Trial making test B	143.2 ± 91.2	≥282	1 (1)
Trial making test B–A	98.8 ± 82.7	≥187	2 (1)
Attentive Matrices (0–60)	50.6 ± 6.2	<31	0 (0)
Stroop time interference effect	23.7 ± 15.0	≥36.92	2 (0)
Stroop error interference effect	1.3 ± 2.0	≥4.24	1 (0)
Verbal fluency on phonemic cue	40.2 ± 12.1	<17.35	0 (0)
Verbal fluency on semantic cue	44.1 ± 9.7	<23.59	0 (0)
Naming (objects) (0–48)	47.2 ± 1.1	≤41.48	0 (0)
Naming (actions) (0–50)	48.1 ± 1.7	≤36.86	0 (0)
Line orientation judgment test (0–30)	22.6 ± 3.8	<19	0 (1)
Unknown face recognition task (0–50)	45.6 ± 4.9	<39	1 (0)
Rey-Osterrieth complex figure copy (0–36)	31.6 ± 2.9	<28.88	1 (0)
Ekman 60 Faces test (0–60)	49.1 ± 6.1	≤37.46	0 (0)
SET Intention attribution (0–6)	4.9 ± 1.2	<2.35	1 (1)
SET Emotion attribution (0–6)	4.3 ± 1.7	<2.21	3 (2)
SET Causal inference (0–6)	4.5 ± 1.4	<2.42	1 (2)
SET global score (0–18)	13.5 ± 3.6	<8.30	2 (1)
IRI Fantasy	17.9 ± 2.4	<11^**^	0 (0)
IRI Perspective Taking	19.1 ± 6.3	<12^**^	1 (4)
IRI Empathic concern	24.4 ± 5.9	<24^**^	6 (1)
IRI Personal distress	20.0 ± 6.2	3 < >27^**^	1 (3)

*SD, standard deviation; UPDRS, Unified Parkinson's Disease Rating Scale; MoCA, Montreal Cognitive Assessment; SET, Story-based Empathy Task; IRI, Interpersonal Reactivity Index*.

* *Based on (Yesavage et al., 1982)*.

** *Due to the lack of Italian normative values, mean, and standard deviation values previously reported (Rankin et al., 2005) were used to derive explorative cut-off scores (mean ± 2 SD for impaired performance; mean ± 1.5 SD for borderline performance)*.

All subjects gave informed consent to clinical evaluation according to the local Ethical Committee. The original study protocol, as well as the telemedicine extension, was approved by the Institutional Review Board (Protocol Number 2019-033).

### Baseline Neuropsychological Assessment

Social cognition abilities have been assessed through different tests evaluating emotion recognition abilities (Ekman 60 Faces Test-Ek-60F) (Dodich et al., 2014) and theory of mind (Story-based empathy task-SET) (Dodich et al., 2015). The SET is a non-verbal task assessing the ability to attribute mental states based on intention (SET-IA) and emotion (SET-EA), as well as the ability to infer causal relationship (i.e., SET-CI). Empathic attitude has been assessed through the Interpersonal Reactivity Index (IRI) (Davis, 1980; Rankin et al., 2005), a 28-item questionnaire administered to caregivers and including both cognitive (perspective-taking IRI-PT, fantasy IRI-F) and affective aspects of empathy (empathic concern IRI-EC, and personal distress IRI-PD). The revised version of the Lubben social network scale (LSNS-R) (Lubben et al., 2006) has been used to assess patients' social network.

Besides, all subjects underwent a standard neuropsychological assessment, including tests evaluating the global cognitive status (Conti et al., 2015), short-term memory [Digit Span forward (Monaco et al., 2013), Corsi block-tapping test (Monaco et al., 2013)], long-term memory [Rey Auditory Verbal List test (Carlesimo et al., 1996), Rey-Osterrieth complex figure recall (Caffarra et al., 2002a)], attention/executive functions [i.e., attentional matrices (Spinnler and Tognoni, 1987), digit span backward (Monaco et al., 2013)], verbal fluency (Carlesimo et al., 1996; Zarino et al., 2014), Stroop task (Caffarra et al., 2002b), trial making test (Giovagnoli et al., 1996), language [i.e., naming (Catricala et al., 2013; Papagno et al., 2020)], visuospatial line orientation judgment test (Benton, 1983), the unknown face recognition test (Benton et al., 1983), and visuo-constructional abilities [Rey-Osterrieth complex figure copy (Caffarra et al., 2002a)].

Caregivers' burden were assessed through the Caregiver Burden Inventory (CBI) (Novak and Guest, 1989). This questionnaire includes 24 items investigating five different dimensions: time-dependence (CBI-TD), developmental (CBI-D), physical (CBI-P), social (CBI-S), and emotional (CBI-E) burden. Higher scores implicate greater burden.

### Telemedicine Assessment

The follow-up assessment was performed through an online platform. The structured interview included questions about patients' health statuses *(“Have you received medical assistance in the last 2 months?” “Have you modified your pharmacological treatment?” “Have you noticed a disease progression?”)* and perceived changes in motor and non-motor symptoms (“*Do you think that your cognitive/motor symptoms are worsened during the lock-down?” “Have you noticed mood changes during the lockdown?”)*. In case of positive answers, subjects were asked to specify. Patients were then asked if they were aware of the COVID-19 emergency, and patients' perceptions were assessed through two questions (“Are you concerned about the effect that COVID-19 may have on your health?” and “How severe do you think the COVID-19 emergency is for the society?”) using a 5-point Likert scale (1: not at all, 5: very much). Together with the structured interview, LSNS-R and CBI scales were also readministered to assess changes in patients' social network and in caregivers' burden.

### Statistical Analyses

We evaluated the percentage of patients with social cognition deficits based on the Italian normative data. Then, in order to assess the effects of social cognition abilities on patients' responses to lockdown measures and to caregivers' well-being, we correlated through Spearman's rank correlation analysis the adjusted performance at social tasks with patients' perceptions of COVID-19 emergency and CBI subscales. Besides, a Mann-Whitney U test has been performed to compare patients' perceptions of the COVID-19 emergency, dividing PD patients according to social cognition impairments, based on literature-defined cut-off scores (Table 1). Finally, CBI and LSNS-R scores were compared through Wilcoxon Signed Ranks Test in order to assess the effect of lockdown measures on caregivers' well-being and patients' social network. Non-parametric statistics were performed due to the small sample size and to non-normal data distribution, evaluated through the Shapiro-Wilk test. Analyses were conducted using IBM SPSS Statistics for Windows v23.0 (Armonk, NY IBM Corp.).

## Results

At the baseline neuropsychological evaluation, 10 patients were classified as cognitively unimpaired, while four patients presented a non-amnestic mild cognitive impairment (Table 1). No patients showed defective performance at the EK-60F test according to the normative data. Emotion attribution, evaluated through SET-EA, was impaired in 3 patients out of 14, with the other two patients obtaining a borderline score. Only two subjects poorly performed in the SET subtask of intention attribution. Notably, in the IRI, five patients showed reduced perspective-taking, while half of the sample was characterized by poor empathic concern. Finally, four patients showed high levels of IRI personal distress, while fantasy was above the cut-off score in all patients. At the follow-up structured interview, three patients felt the disease went faster in the 2 months of lockdown. Four patients reported increased memory difficulties, while increased anxiety was reported in three patients out of 14. A worsening in motor functioning was reported in eight patients out of 14. Three patients received medical assistance related to PD symptoms with subsequent PD-therapy modifications (two therapy reductions secondary to increased levodopa-induced dyskinesia and one increased therapy due to worsening of tremor and bradykinesia). Concerning COVID-19 emergency, all patients were aware of the situation and perceived a high severity for the society (median = 5, IQ range [4.75–5]), despite a low concern for their own health (median = 2, interquartile range [1–3]).

The correlation analysis between COVID-19 perception and social cognition abilities showed a correlation of patients' concerns related to health with patients' perspective-taking abilities (IRI-PT r_s_ = 0.71, *p* = 0.005) and personal distress (IRI-PD r_s_ = −0.72, *p* = 0.004). No significant relationship was found with other social skills. However, IRI-PT was significantly correlated with emotion attribution abilities (SET-EA subscore r_s_ = 0.60, *p* = 0.02) and overall mentalizing abilities (SET-GS r_s_ = 0.55, *p* = 0.04). PD patients with social cognition deficits showed a reduced concern (mdn = 2, interquartile range [1–2]) compared to patients with unimpaired social skills (mdn = 3 interquartile range [2.5–4]) (Mann-Whitney *U* = 40, *p* = 0.04). No significant results were found in the association between judgement of the COVID-19 emergency at the societal level and social skills. MCI and cognitively unimpaired PD patients showed no significant differences in COVID-19 perception (COVID-19 emergency at societal level Mann-Whitney *U* = 19, *p* = 0.9, COVID-19 health-related concern Mann-Whitney *U* = 12, *p* = 0.3), and no significant correlation emerged with the global cognitive status, evaluated through the MoCA score (COVID-19 emergency at societal level r_s_ = −0.17, *p* = 0.5, COVID-19 health-related concern r_s_ = 0.40, *p* = 0.15).

Concerning caregivers' burden, lower perspective-taking abilities were found to be associated at baseline with higher caregivers' burden in the CBI subscales time-dependence (CBI-TD r_s_ = −0.57, *p* = 0.03), Developmental (CBI-D r_s_ = −0.6, *p* = 0.02), and emotional (CBI-E r_s_ = −0.60, *p* = 0.02) burden, as well as in CBI global score (r_s_ = −0.62, *p* = 0.02). Lower mentalizing performance was associated with higher physical (SET-GS: r_s_ = −0.60, *p* = 0.02) and emotional (SET-GS: r_s_ = −0.60, *p* = 0.02) CBI scores. Finally, higher CBI global scores were associated to higher personal distress (IRI-PD r_s_ = 0.56, *p* = 0.04). No other significant correlations emerged. Non-parametric analyses, performed to evaluate significant changes in CBI and LSNS-R, found no significant differences, except for CBI social burden scores, in which caregivers showed a burden reduction compared to the baseline assessment (T = 5, z = −2.1, *p* = 0.04) (Table 2).

**Table 2:** Pre-post comparison of Caregiver Burden Inventory (CBI) and Lubben Social Network Scale-Revised (LSNS-R).

	**Baseline**	**Follow-up**	**Statistics**
**CAREGIVER**			
CBI-global score	12.5 [5.5–30.25]	18 [4.75–29.75]	*z* = −0.5, *p* = 0.7
CBI-time dependence	3 [0–7]	4.5 [1–11]	*z* = −1.4, *p* = 0.15
CBI-developmental	3.5 [0.75–7]	5 [2.25–8.75]	*z* = −0.72, *p* = 0.5
CBI-physical	2.5 [0.75–5.75]	3 [0.25–6.75]	*z* = −0.5, *p* = 0.65
CBI-social	1 [0–7.5]	1.5 [0–2.75]	*z* = −2.1, *p* = 0.04
CBI-emotional	1 [0–3]	1.5 [0–3.5]	*z* = −0.72, *p* = 0.5
**PATIENT**			
LSNS-R	35 [30.7544]	37.5 [28.75–43.5]	*z* = −0.2, *p* = 0.8

*CBI, Caregiver Burden Inventory; LSNS-R, Lubben Social Network Scale-Revised*.

## Discussion

In this preliminary study, we report our experience at ascertaining the implications of COVID-19 in 14 PD patients from our center, and we explored the relationship between social cognition deficits and patients' perceptions of the COVID-19 emergency, as well as the effect of COVID-19 restrictive measures (i.e., lockdown and self-isolation) on caregivers' burden and patients' social isolation.

Overall, the results of the present study possibly suggest that social cognition deficits in PD patients might influence the correct interpretation of the risks related to COVID-19 infection, confirming the first hypothesis of the present study. Despite these results being explorative, we provided evidence of a possible association between lower abilities of perspective-taking and lower concern on the possible effects of COVID-19 on patients' health. Furthermore, a lower concern for COVID-19 was associated with a higher personal distress, which is considered as the negative side of affective empathy (i.e., tendency to feel pain when exposed to the suffering of others). Representing the most primitive precursor of empathy from a developmental point of view, personal distress seems to have an adverse effect in thwarting empathic response rather than enhancing it (Kim et al., 2018). Future studies on larger samples with a comparison group are thus recommended to investigate the effect of social cognition deficits on the perception of the COVID-19 emergency and on the adherence to preventive measures. Recent evidence suggests in fact a higher risk in PD patients for worse respiratory complications after the COVID-19 infection (Fasano et al., 2020; Helmich and Bloem, 2020) and a possible higher mortality rate for older patients with a longer disease duration (Antonini et al., 2020). Thus, it is highly desirable for PD patients to strictly apply to all the required preventive measures to minimize the risk of infection. In our sample, lower empathy and mentalizing ability were also associated with higher caregiver burden. This result is in agreement with previous studies showing that social cognition impairments in PD can significantly affect caregivers' well-being, possibly due to caregivers' lack of awareness of these deficits (Martinez et al., 2018).

On the other hand, despite the risk of negative effects on patients' social isolation and caregivers' burden due to COVID-19 restrictive measures, the lack of significant changes in our sample does not confirm the second hypothesis of the present study (i.e., increase of PD patients perceived social isolation and caregivers burden due to COVID-19 containment**)**. Unexpectedly, caregivers showed a reduction in social burden, possibly suggesting a supporting role of patients' families, also in agreement with the lack of significant changes in patients' social network. This could be partially explained by the Italian sociocultural framework, in which family has a central role in patients' caring (Glaser et al., 2004), or by a reduced burden of daily activities due to the forced lockdown. Overall, the small sample size and the lack of a control group represent the main limitations of the current work, hampering the generalization of the results. Despite finding a good coping to the COVID-19 emergency, studies on larger samples are needed, including patients with different disease severities and with limited access to new technologies. As a matter of fact, while elderly people from rural areas and with low education are those who have the most limited access to new technologies (Marcellini et al., 2007; Poushter, 2016), they are also those who may have been most affected by the social distancing measures. Finally, another limitation of this work is represented by the inability to carefully assess the effects of individual sociodemographic and psychological factors in coping with the COVID-19 emergency. Studies including larger patient samples are required to evaluate the possible effects of behavioral variables (Santangelo et al., 2016; Preis et al., 2017), as well as the role of individual features (e.g., Park et al., 2021), in the interpretation of the COVID-19 emergency. Besides, a more in-depth assessment of patients' perceptions of the risks related to the COVID-19 pandemic is recommended. While no specific questionnaires were available at the time of this preliminary study, the progressive introduction of validated scales and questionnaires (e.g., Cortez et al., 2020) will allow a more detailed evaluation of the cognitive and psychological factors related to the interpretation of the COVID-19 emergency. In conclusion, in this study we provided preliminary evidence in PD of a possible effect of social cognition dysfunctions in interpreting the COVID-19 emergency. Since PD patients represent a vulnerable population for the COVID-19 infection, the development of educational initiatives for both patients and caregivers, possibly converted into telemedicine programs, might help in managing the possible consequences of this new health challenge.

## Data Availability Statement

The raw data supporting the conclusions of this article will be made available by the authors, without undue reservation.

## Ethics Statement

The studies involving human participants were reviewed and approved by Comitato Etico per la sperimentazione con l'essere umano, Università di Trento. The patients/participants provided their written informed consent to participate in this study.

## Author Contributions

LT, CP, MF, AD, and FZ: conception and organization of the project. CM, MF, PN, AG, and EP: acquisition, analysis of data. AD, MF, and CP: data interpretation. AD and CM: first drafting the work. All authors revised the manuscript and provided the approval of the work.

## Conflict of Interest

The authors declare that the research was conducted in the absence of any commercial or financial relationships that could be construed as a potential conflict of interest.
